# Retraction: Capsaicin inhibits migration and invasion via the AMPK/NF-kB signaling pathway in esophagus sequamous cell carcinoma by decreasing matrix metalloproteinase-9 expression

**DOI:** 10.1042/BSR-20190819_RET

**Published:** 2021-04-22

**Keywords:** AMPK, Capsaicin, cell invasion, cell migration, ESCC

This Retraction follows an Expression of Concern relating to this article previously published by Portland Press.

This article is being retracted from *Bioscience Reports* at the request of the Editor-in-Chief and the Editorial Board following receipt of a notification from several readers, alerting the Editorial Board to duplications in Figures 1C, 1D and 5A.

The authors have been contacted by the Journal specifically in regard to the retraction, but have not responded directly to the queries and concerns raised. Given the extent of the issues raised, the Editorial Board stand by the decision to retract the article.

